# Supplementary material for: Quantitative Depth Profiling Using Online-Laser Ablation of Solid Samples in Liquid (LASIL) to Investigate the Oxidation Behavior of Transition Metal Borides
Source: Molecules. 2022 May 18;27(10):3221. doi: 10.3390/molecules27103221 (PMC9147019; doi:10.3390/molecules27103221)
Supplement: Supplementary file 1 [file molecules-27-03221-s001.zip › molecules-1690165-supplementary.pdf]

Table S1. ICP-OES parameters for liquid reference measurements.

**RF-Power**	**1300 W**	
**Radial observation height**	14 mm	
**Cooling gas flow (Ar)**	12 L/min	
**Nebulizer gas flow (Ar)**	0.65 L/min	
**Auxiliary gas flow (Ar)**	0.4 L/min	
**Integration time per replicate**	8 s	
**Replicates per sample**	3	
**Purge pump flow rate**	1.6 mL/min	
**Analysis pump flow rate**	0.8 mL/min	
**Measured Elements**	**Emission line used for quantification [nm]**	**Emission line used for quality control [nm]**
**B**	249.773 (I)	208.893 (I)
**Si**	250.690 (I)	288.158(I)
**Cr**	283.563(II)	284.325(II)

The liquid LOD was calculated by $\frac{3*\sigma}{k}$

Whereas k is the slope of the regression line, and σ is the standard derivation of the blank.

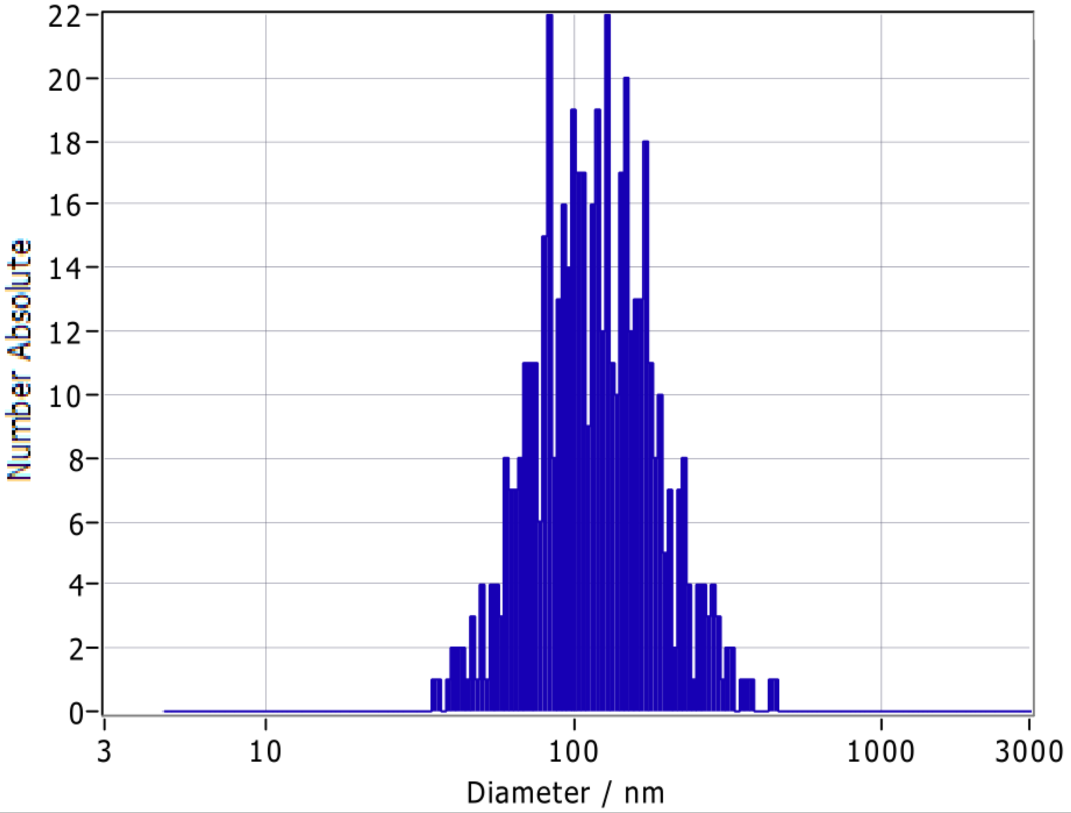

Figure S1. Particle size distribution measurement of the particles generated by online-LASIL
